# An Ecological Momentary Intervention for weight loss and healthy eating via smartphone and Internet: study protocol for a randomised controlled trial

**DOI:** 10.1186/s13063-016-1280-x

**Published:** 2016-03-22

**Authors:** Bastiaan Boh, Lotte H. J. M. Lemmens, Anita Jansen, Chantal Nederkoorn, Vincent Kerkhofs, Gerasimos Spanakis, Gerhard Weiss, Anne Roefs

**Affiliations:** Department of Clinical Psychological Science, Faculty of Psychology and Neuroscience Maastricht University, PO Box 616, 6200 MD Maastricht, The Netherlands; Department of Data Science and Knowledge Engineering, Faculty of Humanities and Sciences, Maastricht University, Maastricht, The Netherlands

**Keywords:** Ecological momentary assessment, Ecological momentary intervention, Obesity, Cognitive behavioural therapy, RCT

## Abstract

**Background:**

Long-term weight loss maintenance is difficult to achieve. Effectiveness of obesity interventions could be increased by providing extended treatment, and by focusing on person-environment interactions. Ecological Momentary Intervention (EMI) can account for these two factors by allowing an indefinite extension of a treatment protocol in everyday life. EMI relies on observations in daily life to intervene by providing appropriate in-the-moment treatment. The Think Slim intervention is an EMI based on the principles of cognitive behavioural therapy (CBT), and its effectiveness will be investigated in the current study.

**Methods:**

A randomised controlled trial (RCT) will be conducted. At least 134 overweight adults (body mass index (BMI) above 25 kg/m^2^) will be randomly assigned to an 8-week immediate intervention group (Diet + Think Slim intervention, *n* = 67) or to an 8-week diet-only control group (followed by the Think Slim intervention, *n* = 67). The Think Slim intervention consists of (1) an app-based EMI that estimates and intervenes when people are likely to overeat, based on Ecological Momentary Assessment data, and (2) ten online computerised CBT sessions which work in conjunction with an EMI module in the app. The primary outcome is BMI. Secondary outcomes include (1) scores on self-report questionnaires for dysfunctional thinking, eating styles, eating disorder pathology, general psychological symptomatology, and self-esteem, and (2) eating patterns, investigated via network analysis. Primary and secondary outcomes will be obtained at pre- and post-intervention measurements, and at 3- and 12-month follow-up measurements.

**Discussion:**

This is the first EMI aimed at treating obesity via a cognitive approach, provided via a smartphone app and the Internet, in the context of an RCT.

**Trial registration:**

This trial has been registered at the Netherlands Trial Register, part of the Dutch Cochrane Centre (NTR5473; registration date: 26 October 2015).

## Background

Obesity is so prevalent that it is considered a pandemic [1]. There are many adverse health effects associated with obesity, such as increased rates of type-2 diabetes, cardiovascular disease, osteoarthritis, obesity-related cancers, and psychological disorders such as depression [2]. As a consequence, in the US, obesity has been associated with an annual medical care cost of US$209.7 billion, corresponding with 20.6 % of total annual spending [3]. In the UK, obesity-related diseases are estimated to add £1.9–2 billion per year to healthcare costs by the year 2030 [4].

So far, behavioural obesity treatment has been largely ineffective at achieving sustained maintenance of weight loss that is required to curb the obesity pandemic [5]. Whereas some degree of initial weight loss is often achieved, it is almost invariably followed by weight regain. Meta-analyses on weight maintenance have shown that diet programmes with or without exercise [6, 7], very-low-energy diets [6, 8], dietary counselling [9], behavioural therapy [10], lifestyle advice [11] and pharmacotherapy [8, 11, 12] mostly lead to weight loss of between 5–9 % of initial body weight, which levels off at around 6 months [8]. After these 6 months, around 50 % of the lost weight (on average) is gradually regained [8, 13]. This indicates that it is important to investigate methods for improving not only immediate weight loss resulting from treatment, but also to prevent weight regain in the long term.

### Increasing the effectiveness of obesity treatment

Two promising factors could increase the effectiveness of behavioural obesity treatment in the long term. Firstly, the high prevalence of weight regain implies that obesity should be considered a chronic condition that requires continuous care [14–19]. It has been shown that providing extended care post obesity treatment (in the form of post-treatment contact sessions with an interventionist) leads to better long-term weight loss maintenance [20]. Observations from the National Weight Control Registry, furthermore, show that people who successfully maintain long-term weight loss continue to consistently monitor their eating behaviour and body weight, engage in physical exercise, and limit caloric intake post weight loss [21, 22]. Secondly, an effective treatment model for obesity should account for interactions between individual (psychological) factors and the food-replete obesogenic environment [1, 18, 23–28].

### Ecological Momentary Interventions

With the advent of mobile technology and the Internet, it has become possible to provide continuous care that can account for person-environment interactions. Ecological Momentary Intervention (EMI) is a framework that combines real-time assessment (Ecological Momentary Assessment, EMA) with treatment. Therefore, EMI allows the provision of (indefinite) care in the natural environment [29]. To accomplish this, assessment and treatment is conducted and provided via a mobile platform, such as a smartphone. The advantage over traditional treatment is that EMI does not necessarily involve therapist contact. This makes treatment more accessible for people who feel reluctant to seek help within a traditional healthcare system. Furthermore, EMI is associated with lower costs [29]. Instead of extensive therapist contact, EMI uses observations of daily life, via for example a smartphone, as input to automatically guide therapy-based techniques and progress. Therefore, EMI is most suitable in combination with a well-defined and structured intervention protocol, such as cognitive behavioural therapy (CBT), which can more easily be automated. So far, only one pilot study has been conducted investigating EMI for obesity, which was aimed at changing the eating behaviour of overweight participants [30]. This study showed that participants’ intake of healthful food increased at the end of the intervention, and that participants considered the intervention acceptable [30]. However, the study relied on providing feedback on EMA data and did not include a treatment protocol. Therefore, more research is necessary to improve insights into the effectiveness of EMI for obesity.

### The Think Slim intervention

The Think Slim intervention is designed to work in conjunction with a calorie-restrictive diet. It consists of (1) an iPhone app-based EMI that will estimate when people will be likely to overeat (‘risky’ moments) and that can intervene at such moments, and (2) a CBT-based intervention aimed at changing patterns of obesity-related dysfunctional thinking and increasing self-esteem. The CBT-based intervention consists of ten online sessions on a computer (computerised CBT; cCBT), and a module in the app. The CBT app module will allow participants to make use of cognitive techniques and reflection practised during the online sessions.

The EMI part of the intervention uses observations in daily life that are obtained via EMA to build a dynamic network of interactions (over time) between psychological variables [31–33]. Specifically, variables with relevance for eating behaviour were included, such as mood [34], physical location and activities [35], food desire strength and specific food desires [36]. Previous research has shown that specific patterns in such a network (i.e. recurring *contingencies* between variables) can be associated with psychological disturbances [33]. For obese participants, contingencies between EMA-assessed eating-related variables modelled in a network were previously found to be more often and more strongly present, leading to a denser network structure compared to non-obese participants [37]. The Think Slim intervention uses contingencies between variables of observations collected in daily life to estimate ‘risky’ moments that indicate when it is likely that someone will overeat. So, by considering network patterns that consistently precede overeating, treatment in the app, in the form of a warning and cCBT-based feedback, can occur prior to an actual eating event.

In addition to estimating ‘risky’ moments, insights into how variables such as mood states, physical locations, and activities are related to eating behaviour will be provided as summarised graphical feedback that is accessible in the iPhone app. Although no data exists for treating obesity, one previous randomised controlled trial found this sort of graphical feedback to be beneficial for treating depression [38].

The second part of the Think Slim intervention is based on the principles of CBT (e.g. [39]). The cognitive theory states that negative emotions and undesirable behaviour are caused and maintained by maladaptive information processing and dysfunctional beliefs. To decrease psychopathology, CBT focuses on identifying and altering the function, content and structure of cognitions, schemas and attitudes associated with undesirable behaviour. Over the course of CBT treatment, patients are guided through several structured sessions aimed at identifying and modifying dysfunctional thinking patterns. Patients are asked to monitor their cognitions and learn to evaluate the validity and utility of these cognitions. Furthermore, they gradually replace unrealistic cognitions with more helpful alternatives.

CBT is one of the most thoroughly supported psychological interventions worldwide [40] and has been found to be effective for a wide range of psychological disorders including, for example, anxiety and depression [41]. Though research in the field of obesity is scarce, it has been suggested that CBT might also be an effective treatment for obesity [24, 42]. Various trials have found support for long-term weight loss maintenance in obese participants [43–45]. However, one trial did not [46]. Furthermore, CBT was found to be effective in reducing dysfunctional cognitions in morbidly obese patients waiting to undergo bariatric surgery [47, 48], and in establishing long-term weight loss in obese participants with binge eating disorder [49].

In the current study, CBT will be provided via online sessions on a computer (cCBT), and via a module in the app. So far, not much is known about the efficacy of cCBT for the treatment of obesity. However, a recent meta-review concluded that cCBT is effective in the treatment of depression [50]. Furthermore, research indicates that cCBT is considered an acceptable intervention by clients [51, 52]. There is also pilot study data indicating that CBT delivered via an app can be as effective as cCBT, with both interventions resulting in a significant decline in depressive symptomatology [53].

### Aims and hypotheses

The current study aims to test the effectiveness of the Think Slim intervention. In a randomised controlled trial, overweight participants will actively follow a diet of their own choice. In addition, they will be instructed to exercise according to their own personal exercise plan. Half of the participants will receive the Think Slim intervention immediately (immediate intervention group). The other half will start with a diet only (diet-only control group). Participants in this latter group will receive the Think Slim intervention afterwards. Whether diet + Think Slim is superior to diet only in the reduction of BMI will be examined together with a set of secondary post-intervention outcomes. Secondary outcomes include (1) self-report questionnaires of dysfunctional thinking, eating styles, general psychological distress, eating disorder pathology, and self-esteem, and (2) network patterns that lead to unhealthy eating. It is expected that over the course of receiving the Think Slim intervention, participants in the immediate intervention group will experience (1) greater reduction in BMI, (2) greater improvements on dysfunctional thinking, self-report questionnaires of eating styles, eating disorder pathology, general psychological symptomatology, and self-esteem, and (3) have less dense network connectivity related to unhealthy eating, as compared to participants in the diet-only control group. Lastly, it is expected that, for both groups taken together, reductions in BMI and improvements in secondary measures will have been maintained at the 3- and 12-month follow-up measurements.

## Methods

### Trial design

**Fig. 1:**
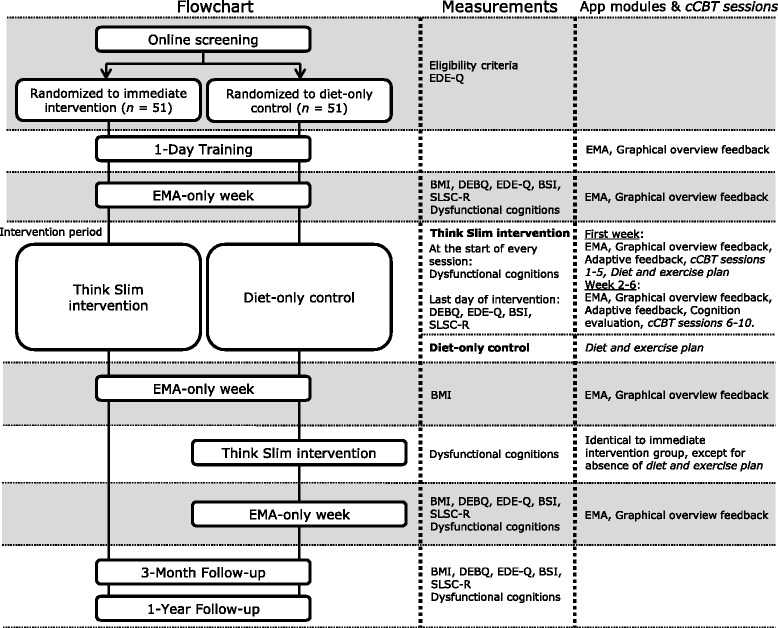
Flowchart for the immediate intervention and diet-only control groups. Note: timing for the 3-month and 1-year follow-up measurements will be calculated from the end of the last Ecological Momentary Assessment (EMA)-only week for both groups

A randomised controlled superiority trial will be conducted, in which 134 overweight (BMI above 25 kg/m^2^) participants will follow a diet of their own choice. In addition, they will exercise according to their own personal exercise plan. Participants are split into two groups. The two groups will run in parallel. The immediate intervention group (*n* = 67) will immediately receive the Think Slim intervention in addition to their chosen diet. During this time, the diet-only control condition group (*n* = 67) will receive no further support. The diet-only control condition group will receive the Think Slim intervention after the immediate intervention group has completed the intervention. The enrolment procedure is graphically shown in Fig. 1. The trial has been approved by the Ethics Committee of the Faculty of Psychology and Neuroscience of Maastricht University (The Netherlands), and is registered at the Dutch Trial Register, part of the Dutch Cochrane Centre (NTR5473). The study will adhere to recommendations of the Consolidated Standards of Reporting Trials (CONSORT) [54].

### Participants

A sample of 134 overweight adults who are recruited in the general Dutch and Belgian population will be included. Applicants will be eligible to participate if they meet the following criteria: age between 18 and 60 years old, BMI above 25 kg/m^2^, sufficient knowledge of the Dutch language, and in possession of an iPhone. Exclusion criteria are: the presence of an eating disorder (as determined with the Eating Disorder Examination Questionnaire, EDE-Q, [55]), the use of appetite-influencing medication (e.g. Suboxone), current treatment for depression, enrolment in other obesity treatments, pregnancy, and a diagnosis of type-2 diabetes.

### Sample size

Sample size was calculated a priori using G*Power [56]. Prior effect size estimates are not available because the current study will be the first to investigate an EMI for weight loss. Power analysis indicated that with 51 participants in each condition, the study is powered at 80.6 % (two-tailed, *α* = 0.05) to detect a standardised medium effect size difference (Cohen’s *d* = 0.5; [57]) in BMI change between the two conditions. To account for an expected 23.5 % attrition rate (derived from attrition rates in EMI studies with ten or more participants, reported in [29]), the aim is to recruit a total of 134 participants.

### Recruitment

Recruitment started in May 2015 and takes place via advertisement (flyers) in healthcare centres and health fairs, online media (e.g. www.proefbunny.nl and www.digi-prik.nl, currently www.onderzoekmachine.nl), social media, and print media outlets. Furthermore, participants from previous studies on eating behaviour who indicated that they were interested in future study participation will be approached for participation. Interested candidates respond to advertisements via e-mail or through the study’s website (www.thinkslim.nl).

### Randomisation and procedure

The study can be divided into five phases: (1) the phase from recruitment to randomisation, (2) EMA-only week 1, in which EMA data on eating behaviour and cognitions will be obtained, (3) the Intervention Phase; in which participants will actively follow a diet, either accompanied by the Think Slim intervention (immediate intervention group) or not (diet-only control group), (4) EMA-only week 2: a second week in which EMA data on eating behaviour and cognitions will be obtained, and (5) the Follow-up (FU) Phase: the period covered by assessment at 3 and 12 months post intervention, and by the reception of the Think Slim intervention by the diet-only control group. In each phase, different modules of the Think Slim app will be active. The flow of the trial, measures and intervention components are graphically depicted in Fig. 1. Each phase will be described in detail below. During the trial, participants are allowed to contact the research staff via e-mail and telephone to discuss any technical or personal issues that might be of influence on study participation. In addition, telephone contact is scheduled prior to the onset of each new phase. Care will be taken to avoid biasing participants during this communication, by referring to the online training and app manual whenever content-related questions arise.

#### Phase 1: from recruitment to randomisation

After initial response to the advertisements, interested candidates will be requested to complete an online screening questionnaire assessing inclusion and exclusion criteria. Eligible participants will receive further study information via e-mail and telephone. This will allow candidates to ask questions and to consider their study participation well before they agree to participate. Participants who are not eligible for participation will be informed via e-mail about the reason(s) for exclusion and will be provided with an opportunity to correspond with the research staff.

After agreeing to participate, participants will provide digital informed consent. Subsequently, participants will be contacted to plan a 1-day technical training via the app shortly afterwards, and will be sent a manual for the app via postal mail and via e-mail. The purpose of the technical training is to ensure that the app is working and to minimise the influence of differences in technical aptitude.

#### App functionality during the technical training

During the technical training, the EMA module and Graphical Overview Feedback module of the app will be active. The EMA module of the Think Slim app will be used to obtain real-time data of eating-related variables. Items that are part of the EMA questionnaires include measures of food desire strength, specific food desires, emotions, cognitions, locations, activities, social company and food consumption. During the training, EMA will be performed at two separate instances: (1) eating events (event sampling), and (2) eight times pseudorandomly throughout the day (time-contingent sampling). Sampling of an eating event will be initiated by the participants themselves, whereas time-contingent sampling points will be generated by the app. Note that cognitions and food consumption will only be assessed during eating event sampling. Otherwise the eating event samples and time-contingent samples are the same. The first time-contingent sample of the day will also assess sleep duration and quality. Beverage consumption will be assessed in a separate assessment, which will occur prior to bedtime.

In addition to the EMA module, graphical overview feedback will be accessible in a separate section of the app. The feedback will represent pie charts of the activities and locations assessed at eating event samples by the participant, separately for foods that are considered healthy and unhealthy. In addition, a line graph of desire strength and strength of various emotions, presented relative to time, will be available. The graphical overviews will continuously be updated, as soon as new EMA data is entered into the app. To generate the overviews, at least one eating event sample is necessary.

#### Randomisation

After completing the technical training, all participants will be contacted by the researchers to discuss possible issues that occurred during the training. Subsequently, randomisation will take place. Participants will be randomly assigned to either the immediate intervention group or the diet-only control group using computer-generated randomisation via Research Randomizer (www.randomizer.org). More specifically, randomisation is pre-stratified by age group (18–30; 31–40; 41–50; 51–60) and gender. For each age group, separately for male and female participants, each participant will be assigned a randomly generated number. Half of the participants with the largest randomly generated numbers will be assigned to the immediate intervention group, whereas the other half will be assigned to the diet-only control group. Note that participants who indicate as being unavailable during a specific time period, due to for example holidays, will be manually allocated to the condition that matches with their availability. Blinding participants and researchers is not possible in the current setup.

#### Phase 2: EMA-only week 1 (week 1)

The trial starts with an EMA-only week. At the start of this week, all participants (immediate intervention group and diet-only control group) will fill out a baseline assessment consisting of several self-report questionnaires (specified below, under ‘Measures’) about eating styles, eating disorder pathology, general psychological distress, self-esteem and dysfunctional thinking. In addition, participants will obtain an independent measure of their BMI. No further intervention will take place.

#### App functionality during the first EMA-only week

During the entire week, the EMA module and Graphical Overview Feedback module of the app will be active, identical to the technical training.

#### Phase 3: Intervention Phase (weeks 2–7)

After the first EMA-only week, the Intervention Phase starts. This phase has a total duration of 6 weeks (weeks 2–7).

#### Diet

During the Intervention Phase, all participants (immediate intervention group and diet-only control group) are asked to actively follow a calorie-restrictive diet of their own choice. Supervision of the diet by a dietician or healthcare professional is not allowed. Instead, all participants select a diet coach (friend or family member). Participants will also develop an exercise plan. General information about dieting and exercising is provided as part of two online sessions in the first week of the Intervention Phase. At the end of the first week, participants are asked to finalise their choice. Aside of the two online sessions, no further information about selecting a diet is provided.

#### Cognitive intervention (for immediate intervention group only)

The cognitive intervention will be aimed at changing obesity-related dysfunctional thinking and at increasing self-esteem. It consists of ten individual online sessions on a computer (cCBT), and a module in the app. The computer sessions are included because CBT requires explanation of the theoretical model and therapeutic concepts that are too comprehensive to provide via an iPhone app due to screen size limitations that prevent a convincing digital presentation of the intervention. Therefore, the online sessions provide participants with the necessary information on the cognitive model and cognitive techniques, whereas the app is suitable to practise these skills in daily life.

There are a total of ten online sessions of approximately 25 minutes that will be delivered via an in-house web-environment for online therapy and questionnaire research (SOTO). To start each session, participants receive an e-mail with a link to the SOTO web-environment. The sessions are based on several CBT for weight loss protocols [45, 58, 59]. They are designed to be provided in addition to a standard calorie-restrictive diet. Table 1 contains a brief summary of each session’s contents. In the first week of the Intervention Phase, five sessions will take place. In these sessions, participants will determine reasons for losing weight, set dietary goals, choose a diet, make an exercise plan, and make a list of techniques to distract from food craving. All information except the choice of diet and exercise plan will be accessible for review in the app. Furthermore, participants will be familiarised with the cognitive model and basic cognitive techniques. They will also learn how to identify and evaluate dysfunctional cognitions, and how to replace these with alternative cognitions that are more helpful towards eating behaviour and dieting. For the first week, all five sessions will need to be completed at the end of the week. Participants who do not complete all five sessions will be considered dropouts. Participants will be sent reminder e-mails in case they lag behind and will be contacted by the research staff in case they are close to being considered dropouts.

**Table 1:** Summary of the contents of the computerised cognitive behavioural therapy (cCBT) sessions

#	Theme	Description
1.	Introduction	Information on weight-gain and weight-loss Introduction of the cognitive model and the concept of dysfunctional thinking Information on how to select a diet, diet coach and exercise plan^a^
2.	Motivation	Personal reasons for losing weight^b^ Realistic weight loss goals^b^ Identifying and responding to sabotaging cognitions about dieting Personal list of sabotaging cognitions about dieting^b^
3.	Coping with food desire	Information on the difference between hunger and food desire Tips on how to deal with food desire Personal list of distraction techniques^b^
4.	Identifying dysfunctional cognitions	Identifying dysfunctional cognitions about eating Introducing the concept of dietary temptations Instruction on how to do this in the app
5.	Responding to dysfunctional cognitions	Introduction of socratic questioning Information on how to construct functional alternative cognitions Instruction on how to do this in the app Choosing a diet and exercise plan^a,c^
6.	Thinking errors	Summary of basic CBT skills Introduction of concept of thinking errors Instruction on how to do this in the app
7.	Interim evaluation	Evaluation on progress so far^c^ Increasing motivation Learning to compliment oneself
8.	Self-esteem: body	Positive body exposure
9.	Self-esteem: person	Designing a positive portrait of the self^c^
10.	Evaluation and relapse prevention	Summary of intervention^c^ Evaluation of intervention Creating a relapse prevention plan^c^

Note: the content of these sessions is derived from several cognitive behavioural therapy (CBT) protocols [45, 58, 59].

^a^component is completed by immediate intervention and diet-only control groups simultaneously; ^b^information is transferred to the app for review; ^c^information will be sent to participants by e-mail; in each session, the level of dysfunctional thinking will be assessed by believability ratings (visual analogue scales (VASs)) of nine frequently occurring dysfunctional cognitions about eating

From the second week until the last (week 7) of the Intervention Phase, participants will complete additional cCBT sessions at the end of each week (sessions 6–10). In these sessions, cognitive skills are reviewed, and participants are encouraged to stay motivated. Furthermore, self-esteem is addressed in two sessions, focusing on the body and on personality characteristics. In the final session special attention is paid to evaluation and relapse prevention. All online cCBT sessions will be completed without therapist involvement. Participants are allowed to lag behind one session, and will be considered dropouts if more than one session is not completed on time. At the beginning of each session, dysfunctional thinking will be assessed via a self-report questionnaire. Participants in the diet-only control group will not receive any cognitive intervention during the Intervention Phase.

#### App functionality during the Intervention Phase

Participants in the diet-only control group will not be able to use the app during the Intervention Phase. For these participants, all app functionality will be blocked. Instead, the app will display the number of days remaining until the end of the waiting period. Eight days before the end of the waiting period, the app will send a notification informing participants that the end of the waiting period is near.

For participants in the immediate intervention group, app functionality in the first week of the Intervention Phase will consist of the EMA module and the Graphical Overview Feedback module, similar to the training and the first EMA-only week. In addition, the Adaptive Feedback module will be active. The Adaptive Feedback module provides participants with feedback if they are at risk for overeating. The feedback message consists of a generic warning and a behavioural advice. Detection of a participant’s ‘risky’ moments is based on the answers provided by the participant on the EMA items. To estimate when the participant is likely to eat something unhealthy in the time-period directly following a completed time-contingent sample, the sample data is compared to a set of allocated pre-existing rules that indicate what combinations of states of variables (e.g. scoring high on desire + being at home + feeling bored + feeling calm) are predictive of unhealthy eating. If there is a match between the time-contingent sample and one of the rules, the participant receives adaptive feedback messages via the Think Slim app.

To provide adaptive feedback, the Think Slim app uses 65 rules that were obtained via a computational decision-tree algorithm (adapted for EMA data) used on a dataset from a previous EMA study that had an almost identical EMA protocol (Boh B, Jansen A, Clijsters I, Nederkoorn C, Lemmens L, Spanakis G, & Roefs A. Indulgent thinking. Ecological Momentary Assessment of Overweight and Healthy-weight Participants’ Cognitions and Emotions, submitted). Each of the 65 rules represents states of eating-related variables that were most frequently reported together right before a healthy or unhealthy eating event, as reported by participants of that study. More specifically, participants were divided to six groups according to their rule-triggering occurrence and each group is represented by a set of rule that describes 80 % of the eating behaviour of participants in the group (thus removing rules with low occurrence and keeping only those with high predictive value). For the current study, each participant will be allocated one of the six rule-triggering groups based on EMA data from the first EMA-only week. At the end of the third week of the Think Slim intervention, EMA sample data from the first 3 weeks of the Intervention Phase will be used to update the allocation of participants to the rule-triggering groups.

From the second week of the Intervention Phase until the end of the intervention, the app functionality will be further expanded. First, participants will be instructed to also assess moments of dietary temptation (event sampling) in the app. Participants will be instructed in an online cCBT session about what qualifies as a dietary temptation. The EMA items assessed during moments of dietary temptations are similar to those assessed during an eating event, except for the food consumption item: food desire strength, specific food desires, emotions, location, company, and activities. Second, the Cognition Evaluation module will be active. The purpose of this module is to identify and evaluate dysfunctional cognitions in daily life. Table 2 contains an overview of the cognitions included in the app and whether or not these are considered dysfunctional. The module designed to work in conjunction with the online cCBT sessions: the module requires knowledge of the basic cognitive skills that are provided in online sessions 1–5.

**Table 2:** Cognition types included in the Think Slim app and the categories they belong to

Category	Cognition type	Example
Neutral	Description of an eating event	‘Time for breakfast!’
	Hunger	‘I am very hungry, time to eat.’
	Desire and taste	‘I really want this chocolate bar.’
	Energy needed	‘I won’t have time to eat later.’
Functional	Healthy intention	‘Apples are good for me.’
	Successful control	‘Everyone’s eating cake, but I’m going for a pear.’
Dysfunctional	Negative emotions	‘I feel awful, maybe this helps.’
	Positive emotions	‘I’m trying to relax with snacks.’
	Social activities and pressure	‘Everyone’s eating cake. I will join them.’
	Reward	‘I deserve this after all the hard work!’
	Control failure	‘I just can’t resist.’
	Other dysfunctional cognitions	‘Coffee should go together with a cookie.’

Note: these cognition types are based on previous research (Boh B, Jansen A, Clijsters I, Nederkoorn C, Lemmens L, Spanakis G, & Roefs A. Indulgent thinking. Ecological Momentary Assessment of Overweight and Healthy-weight Participants’ Cognitions and Emotions, submitted)

When a dysfunctional cognition is selected by a participant in the Cognition Evaluation module of the app during an eating event or dietary temptation, the participant is encouraged to critically evaluate the selected cognition via socratic questions, which aim to guide the evaluation process. For the ease of evaluation, the cognition is presented in a conditional (if-then) format. After the evaluation, the participant is asked to aggregate the information obtained in the evaluation section and use this information to formulate a helpful and realistic response to the initial dysfunctional cognition. Subsequently, the believability of the new cognition is rated. To conclude, the participant is asked to take another look at the initial cognition and rate believability again. The idea is that after evaluation, the believability – and therewith the influence – of the initial cognition decreases. All evaluated cognitions remain accessible in the app for future reference. If a functional or neutral cognition is selected in the app by a participant, the app also provides feedback. In case of an eating event, this feedback will be a generic message (e.g. ‘Enjoy your meal’.). In case of a dietary temptation, an appropriate cCBT-based feedback message will be provided to the participant. This feedback message will refer back to what was learned during the cCBT sessions if the selected cognition is considered dysfunctional.

Lastly, at the last day of week 7, a post-intervention assessment will take place during which participants will fill in self-report questionnaires identical to the baseline assessment. Participants are also instructed to obtain a second measurement of weight during week 8, under identical circumstances as during the first EMA-only week.

#### Phase 4: EMA-only week 2 (week 8)

After the Intervention Phase, all participants (immediate intervention group and diet-only control group) enter a second EMA-only week. This week is identical to the first EMA-only week for all participants.

#### Phase 5: Follow-up (FU) Phase

At the end of the second EMA-only week (week 8), participants in the immediate intervention group will be debriefed about the purpose of the trial. Furthermore, they will receive a monetary reward of €50, in coupons. After that, their FU Phase starts. The FU Phase has a total duration of 12 months. Participants are invited to fill out questionnaires (identical to the baseline and post-intervention assessments) and to obtain independent BMI measurements at two time-points during this period: at 3 and 12 months post intervention. They will receive an additional €20, in coupons, per completed FU measurement.

For the diet-only control group, the procedure after the second EMA-only week (week 8) is different. Participants allocated to this group will now receive the Think Slim intervention (weeks 9–14), followed by a third EMA-only week (week 15) and a debriefing. The content and procedure of the Think Slim intervention received by the diet-only control group is identical to that received by the immediate intervention group, except that information concerning the diet and exercise plan is not included. Furthermore, the third EMA-only week is identical to the other two EMA-only weeks. After that, the 12-month FU Phase starts for this group. The procedure of the FU Phase for the diet-only control group is identical to the procedure of the immediate intervention.

### Measures

Several instruments will be used to assess the effects of the Think Slim intervention on BMI and secondary outcomes. Data are obtained by means of self-report instruments, measures taken by healthcare professionals, and EMA data. Figure 1 depicts when each measure is obtained during the flow of the study.

#### Body mass index

Participants are instructed to obtain multiple measurements of body weight and one measurement of height. These measurements are used to calculate BMI. To ensure objectivity, participants are asked to obtain the measurements via a healthcare professional (e.g. a general practitioner, or a physical therapist), or at the university (Think Slim research staff). Participants are further requested to obtain all measurements under identical circumstances (i.e. the same general practitioner or physical therapist).

#### Patterns of eating behaviour

To gain insight in (changes in) eating patterns, EMA data obtained during the first EMA-only week and second EMA-only week will be investigated.

#### Dysfunctional thinking

Dysfunctional thinking will be measured by believability ratings of nine frequently occurring dysfunctional cognitions about eating (e.g. ‘If everyone is eating a snack, then I should participate’.). Participants are instructed to read each statement carefully, and to answer the question ‘How much do you believe in this cognition?’ by scoring believability on a visual analogue scale (VAS) ranging from 0 to 100; anchors ‘not at all’ and ‘very much so’. Cognitions are selected based on previous research (Boh B, Jansen A, Clijsters I, Nederkoorn C, Lemmens L, Spanakis G, & Roefs A. Indulgent thinking. Ecological Momentary Assessment of Overweight and Healthy-weight Participants’ Cognitions and Emotions, submitted) and observations in clinical practice.

#### Eating styles

The Dutch Eating Behaviour Questionnaire (DEBQ; [60]) will be used to measure eating styles and consists of 33 items across three subscales: external eating, emotional eating and restraint. Each item of the DEBQ is rated on a five-point Likert scale ranging from 1 (seldom) to 5 (very often). Item scores for the three subscales will first be summed separately to obtain an overall score per subscale. Then, for each subscale, the overall score will be divided by the number of subscale items to obtain the final average score per subscale. A high score on one of the subscales indicates a higher tendency to display the respective eating style. The DEBQ was previously found to be internally consistent overall, *α* = 0.79 [61]. The subscales emotional eating (*α* = 0.89), external eating (*α* = 0.078) and restrained eating (*α* = 0.92) were also found to be internally consistent [62].

#### Eating disorder pathology

The Dutch version of the Revised Eating Disorder Examination Questionnaire (EDE-Q; [55]; Dutch translation) will be used to measure eating disorder pathology. This self-report questionnaire consists of 22 items assessing eating pathology, and 6 items assessing diagnostic behaviours such as use of laxatives and purging. The items are scored across four subscales: restraint, weight concerns, shape concerns, and eating concerns. The eating pathology items of the EDE-Q are each scored on a seven-point Likert scale. The diagnostic behaviour items are scored by participants indicating the number of times in the past 28 days that the behaviour occurred. The test-retest reliability of the English version of the EDE-Q was found to be between *r* = 0.66 and *r* = 0.94 for each of the subscales, whereas the internal consistency of the EDE-Q subscales was found to be good, ranging from *α* = 0.70 to *α* = 0.93 [63].

#### General psychological symptomatology

The Brief Symptom Inventory (BSI; [64]: Dutch translation by [65]) will be used to assess general psychological symptomatology, and consists of 53 items. The BSI has nine subscales assessing different forms of psychological distress. Items of the BSI are scored on Likert scales ranging from 0 (not at all) to 4 (very much so). Scores for each subscale are calculated by averaging scores on the individual items belonging to that subscale. The total score for the BSI is calculated by summing all individual item scores. The BSI subscales were found to have satisfactory internal consistency, ranging from *α* = 0.71 to *α* = 0.88 [66]. In addition, the test-retest reliability is high, with the lowest score reported at *r* = 0.68 [66].

#### Self-esteem

The Self-liking and Self-competence Scale Revised (SLSC-R; [67]: Dutch translation by [68]), will be used to assess self-liking and self-competence as dimensions of self-esteem, and consists of 16 items, 8 for each dimension. Items are rated on five-point Likert scales ranging from 1 (strongly disagree) to 5 (strongly agree). Overall scores per dimension are obtained by summing scores of individual items belonging to each dimension, and can range between 8 and 40. Higher scores indicate more self-competence and self-liking than lower scores. Internal consistency of the subscales of the Dutch translation of the SLSC-R was found to be good, ranging from *α* = 0.78 to *α* = 0.98 [68]. Split-half reliability for the subscales was also found to be good, ranging from *r* = 0.75 to *r* = 0.92.

#### Acceptability and compliance

Acceptability of the Think Slim intervention will be measured using a questionnaire that was developed for the current study. This questionnaire includes items on user-friendliness and perceived usefulness of the Think Slim intervention. Compliance with the EMA module in the app will be measured daily during the study by calculating how many time-contingent samples were completed by participants relative to the total number of time-contingent samples that were sent by the app, and by monitoring how often the Cognition Evaluation module of the app was used.

### Data analysis

Participant’s flow from screening to randomisation will be mapped out. Furthermore, pre-treatment characteristics of the sample will be explored, and baseline differences between conditions will be examined in terms of size and clinical importance. Distributions of outcome variables will be inspected via histograms and boxplots. After that, intervention- and study-compliance will be determined, followed by an evaluation of participants’ satisfaction with the Think Slim intervention.

To test the effectiveness of the Think Slim intervention, 2 (group: immediate intervention versus diet-only control group) × 2 (time: pre versus post) analyses of variance (ANOVAs) will be run for the set of dependent variables (i.e. BMI, dysfunctional thinking, scores for questionnaires on eating styles, eating disorder pathology, general psychological symptomatology, and self-esteem). Long-term effects of the Think Slim intervention on the set of dependent variables will be examined using one-way repeated-measures ANOVAs collapsing over group (i.e. for all 102 participants), with four levels: pre-Think Slim, post-Think Slim, 3-month follow-up and 12-month follow-up. All analyses will be performed according to the intention-to-treat principle [69] and the completers principle. Lastly, time-lagged multilevel network analyses will be conducted on data obtained during the pre- and post-Intervention Phase EMA-only weeks, to assess changes in patterns of data that result in unhealthy eating [31, 37]. Changes in network connectivity between the pre- and post-Intervention Phase networks will be compared between both conditions. Of particular interest are changes in network connectivity and density related to unhealthy eating, in terms of graph analysis measures such as indegree, outdegree, betweenness, and centrality of network nodes.

## Discussion

Whereas short-term weight loss is often achieved in existing interventions for obesity, this weight loss is almost invariably followed by weight regain. EMI may help reduce weight regain because it allows indefinite treatment and can assist obese people online (i.e. at moments of weakness). This article presented the design of an RCT comparing the effects of the Think Slim EMI with a diet-only control condition, in terms of (1) weight loss, (2) changes in self-report measures of dysfunctional thinking, eating styles, eating disorder pathology, general psychological symptomatology and self-esteem, and (3) changes in eating patterns. The trial includes follow-up measurements of weight at 3 months and at 12 months. The current study is, to the authors’ knowledge, the first to test a completely online and app-based protocol of cCBT for obesity, with no therapist involvement. With the current study, an effort is undertaken to improve understanding of long-term weight loss and the influence of cognitions and behaviour on achieving such long-term weight loss. With this we aim to contribute to the improvement of interventions for eating behaviour and obesity.

### Trial status

Recruiting.

## Abbreviations

ANOVA: analysis of variance
BMI: body mass index
BSI: Brief Symptom Inventory
CBT: cognitive behavioural therapy
cCBT: computerised cognitive behavioural therapy
DEBQ: Dutch Eating Behaviour Questionnaire
EDE-Q: Eating Disorder Examination Questionnaire
EMA: Ecological Momentary Assessment
EMI: Ecological Momentary Intervention
RCT: randomised controlled trial
SLSC-R: Self-liking and Self-competence Scale Revised
